# Dynamic enhancement patterns of intrahepatic cholangiocarcinoma in cirrhosis on contrast-enhanced computed tomography: risk of misdiagnosis as hepatocellular carcinoma

**DOI:** 10.1038/srep26772

**Published:** 2016-05-26

**Authors:** Rui Li, Ping Cai, Kuan-sheng Ma, Shi-Yi Ding, De-Yu Guo, Xiao-Chu Yan

**Affiliations:** 1 Dept. Hepato-biliary-Pancreatic Surgery, Southwest Hospital Affiliated to Third Military Medical University, P. R. China; 2 Dept. Radiology, Southwest Hospital Affiliated to Third Military Medical University, P. R. China; 3 Dept. Pathology, Southwest Hospital Affiliated to Third Military Medical University, P. R. China.

## Abstract

This study aimed to assess the features of intrahepatic cholangiocarcinoma (ICC) at computerized tomography (CT) and verify the risk of misdiagnosis of ICC as hepatocellular carcinoma (HCC) in cirrhosis. CT appearances of 98 histologically confirmed ICC nodules from 84 cirrhotic patients were retrospectively reviewed, taking into consideration the pattern and dynamic contrast uptake during the arterial, portal venous and delayed phases. During the arterial phase, 53 nodules (54.1%) showed peripheral rim-like enhancement, 35 (35.7%) hyperenhancement, 9 (9.2%) hypoenhancement and 1 (1.0%) isoenhancement. The ICC nodules showed heterogeneous dynamic contrast patterns, being progressive enhancement in 35 nodules (35.7%), stable enhancement in 28 nodules (28.6%), wash-in and wash-out pattern in 15 nodules (15.3%) and all other enhancement patterns in 20 nodules (20.4%). There were no significant differences in the dynamic vascular patterns of ICC according to nodule size (*p* > 0.05). ICC in cirrhosis has varied enhancement patterns at contrast-enhanced multiphase multidetector CT. Though the majority of ICC did not display typical radiological hallmarks of HCC, if dynamic CT scan was used as the sole modality for the non-invasive diagnosis of nodules in cirrhosis, the risk of misdiagnosis of ICC for HCC is not negligible.

Intrahepatic cholangiocarcinoma (ICC) originating from biliary epithelial cells is the second most common primary cancer of the liver after hepatocellular carcinoma (HCC) in patients with cirrhosis. The incidence and mortality of ICC have been rising internationally^1^,^2^,^3^,^4^ and some studies have shown an association of ICC with cirrhosis^5^,^6^,^7^. The prognosis of ICC is dismal with respect to HCC and the treatment modalities of ICC and HCC are of much difference^8^. In this context, differential diagnosis between ICC and HCC in cirrhotic patients is very important for clinical management. According to practice guidelines of the American Association for the Study of Liver Diseases (AASLD) and the European Association for the Study of the Liver (EASL), radiological diagnosis of HCC in patients with cirrhosis relies on either magnetic resonance imaging (MRI) or contrast enhanced computerized tomography (CT) in the context of a sequential algorithm^9^,^10^, demonstrating the hallmark of intense arterial uptake followed by venous or delayed phase washout. Contrast-enhanced computed tomography is now considered a standard of care for the radiological diagnosis of HCC, this modality, however, is not validated to exclude ICC in cirrhosis, robust studies on diagnostic accuracy of contrast-enhanced CT for ICC in cirrhotic liver are not available. Up to now, there were only few reports on CT appearances of ICC in cirrhosis with limited number of patients,, and more importantly, the results of these previous studies are contradictory^11^,^12^,^13^. Therefore, the aim of this study was to assess of the enhancement features of ICC on contrast-enhanced multidetector CT in cirrhotic patients in relation to size and dynamic enhancement pattern of ICC nodules, and with special emphasis on differential diagnosis between ICC and HCC in a relatively larger number of patients.

## Materials and Methods

### Patients

This retrospective study was approved by the ethics committee of Southwest hospital and the requirement for informed consent waived. The study protocol conforms to the ethical guidelines of the 1975 Declaration of Helsinki. Through the review of our institute database between January 2005 and July 2015. We enrolled patients with: (1) the diagnosis of ICC had to be pathologically proven through assessment of biopsy or surgical specimen, excluding mixed hepatocellular-cholangiocarcinoma; (2) histologically confirmed cirrhosis; (3) abdominal contrast-enhanced CT performed before biopsy or resection; (4) No systemic chemotherapy or targeted therapy prior to CT scan.

### Liver histology

Histological diagnosis was performed on surgically resected specimens and/or by biopsy. Ultrasound guided biopsy was performed using a 18 gauge needle (Bard Peripherals Vascular Inc, Tempe, Arizona 85281, USA) within the nodule and the surrounding liver parenchyma. Specimens were routinely processed, stained with hematoxylin and eosin and immunohistochemically for biliary differentiation markers if necessary. Liver sections were examined by experienced liver pathologists who were unaware of clinical and radiological exams. Final pathological diagnosis was made in consensus by experienced pathologists with over 25 year of liver pathology (XCY and DYG).

### Serum tests

Routine exams included blood cell count and serum chemistries were measured by standard laboratory procedures. Serum alpha-fetoprotein (AFP, Elecsys, Roche Diagnostics GmbH Sandhofer Strasse, Mannfeim, Germany; normal, ≤20 ng/ml) was tested in all patients. Carbohydrate antigen 19-9 (Ca 19-9) was also tested in all cases. Serum hepatitis B surface antigen, antibody to hepatitis surface antigen, antibody to hepatitis delta virus and antibody to hepatitis C virus were also tested in all patients. To categorize patients in terms of etiology, a complete medical history, including alcohol intake, smoking habits and other risk factors for liver disease like hemochromatosis, non-alcoholic steatohepatitis, autoimmunity, and diabetes were also recorded.

### CT scan

CT-scans were performed with a multidetector-row helical quadruplephase (i.e., unenhanced, hepatic arterial, portal, and delayed phases) CT scanner (MDCT; Definition, Siemens Medical Systems, Erlangen, Germany). First, an unenhanced scan was obtained through the liver. Next, after intravenous infusion of 80–100 ml of a nonionic iodine-containing contrast agent (ultravist 370, Scherning AG, Berlin, Germany) using a power injector (Stellant CT Injection System, Medrad, Indianola, Pennsylvania) at a rate of 4 ml/sec, contrast-enhanced scans were obtained in arterial phase with bolus test trigger for optimal characterization of focal hepatic lesions. Data acquisitions were obtained through the whole liver in a craniocaudal direction during a single breath-hold helical acquisition for 4–6 sec with 5 mm slice thickness and 0.5 s rotation time. The acquisition of the arterial phase was automatically started 4 s after the arrival of contrast agent in the aorta. The start of acquisition sequences was 60 s for the portal venous phase and 180 s for the delayed phase from the beginning of contrast agent injection.

CT findings were evaluated blindly by 2 radiologists with over 20 year experience of liver radiology (PC and SYD) who were blinded to the clinical and histological results. All imaging films were independently evaluated by both radiologists whereas discrepant diagnoses were jointly re-evaluated to reach a final consensus.

### Categorization of enhancement patterns at CT

After intravenous contrast administration, the enhancement through each of the different phases was registered as follows: (1) hyperdense: increased density involving predominant parts (>50%) of the lesion cross-section area compared to the surrounding liver parenchyma^13^; (2) peripherally hyperdense: increased density limited to the periphery of the lesion, resembling a rim-like pattern; (3) isodense: same density as the surrounding liver parenchyma; (4) hypodense: lower density compared to the liver parenchyma involving predominant parts (>50%) of the cross-sectional area of the tumor excluding peripheral rim-like enhancement. Dynamic pattern of enhancement was defined according to the combination of contrast enhancement in the different phases of the study (arterial, portal venous, delayed-venous), as follows: (1) stable enhancement: the nodule enhancement is unmodified from the arterial to the portal venous and delayed phases; (2) progressive contrast enhancement: the nodule enhances progressively over time, reaching maximal intensity in delayed phases; (3) “wash-in and wash-out” enhancement pattern: intense hyperdense during the arterial phase followed by hypodense in the portal and/or delayed venous phases; (4) all other patterns. This classification was modified from Iavarone *et al*.^14^.

### Statistical analysis

Quantitative variables, such as tumor size, age, serum levels of AFP, and Ca19-9, were expressed as median and range. Qualitative variables like etiology of liver disease, Child-Pugh score, and number of nodules, were expressed as count and proportions. Differences in signal intensity in baseline and post-contrast sequences and in dynamic enhancement pattern according to nodule size were evaluated by the Chi-squared test/Fisher’s exact test for categorical variables. A *P* value of less than 0.05 was considered statistically significant. Statistical analysis was performed using the SPSS 12.0 software package (SPSS Inc, Chicago, IL).

## Results

### Characteristics of patients

This search yielded a total of 98 histologically proven ICC in 84 patients with cirrhosis (66 men; mean 50.6 ± 10.9 years). Characteristics of patients are summarized in Table 1. Etiology of cirrhosis was HBV infection in most patients (85.7%). Near half of the patients were cigarette smokers. Mean AFP level was 148.49 ± 732.88 ng/ml and elevated in 19 patients (22.6%). Mean CA19-9 was 104.59 ± 166.19 U/ml and elevated in 43 patients (51.2%). Most patients (89.3%) had a single ICC at diagnosis. In patients with multiple nodules, each nodule was located in a different segment thereby excluding the diagnosis of peripheral satellite. Thirty–eight patients (33.3%) were under semi-annual surveillance at the time of first diagnosis.

### CT scan features

The size of ICC detected by CT at diagnosis was 5.6 ± 2.7 cm. Twenty one nodules (20.4%) were ≤ 3.0 cm while 77 (78.6%) nodules >3.0 cm. Hepatic capsular retraction was revealed in 11cases (13.1%). Intrahepatic biliary dilatation was observed in 17 patients (20.2%). Nine patients had malignant portal veins thrombus (10.7%) and 12 patients had regional lymphadenopathy (14.3%). Hepatic heamangioma was observed in 1 patient (1.2%) and hepatic cyst noted in 5 patients (6.0%). Intrahepatic lithiasis was seen in 1 patient (1.2%).

The contrast appearance of ICC during different vascular phases is summarized in Table 2.

### Dynamic enhancement patterns at CT

**Figure 1:**
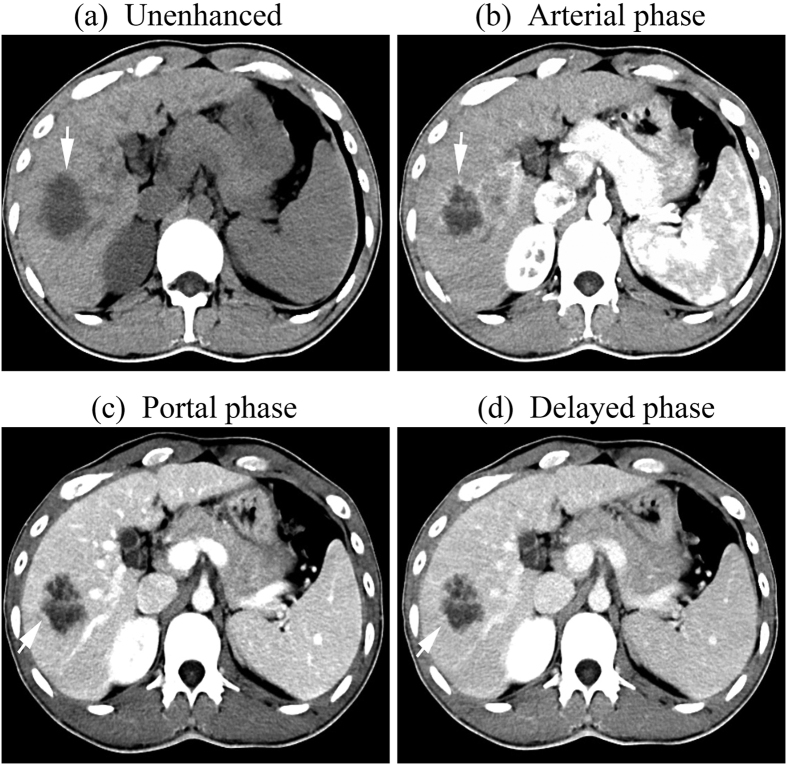
tif CT images of a 32-year-old man with a 4.2-cm –diameter ICC displaying stable contrast enhancement pattern. (**a**) unenhanced, (**b**) Arterial, (**c**) portal, and (**d**) delayed phases. After intravenous contrast administration, the nodule shows stable peripheral rim-like enhancement (arrow).

**Figure 2:**
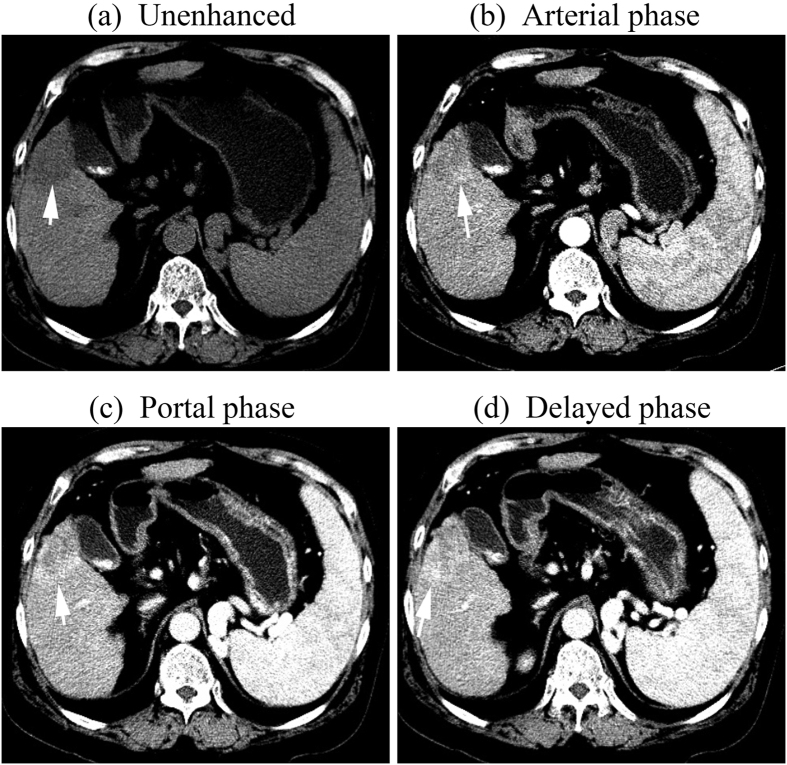
tif CT images of a 75-year-old man with a 3.8-cm –diameter ICC displaying progressive contrast enhancement pattern. (**a**) unenhanced, (**b**) Arterial, (**c**) portal, and (**d**) delayed phases. After intravenous contrast administration, the nodule shows progressive centripetal enhancement (arrow).

**Figure 3:**
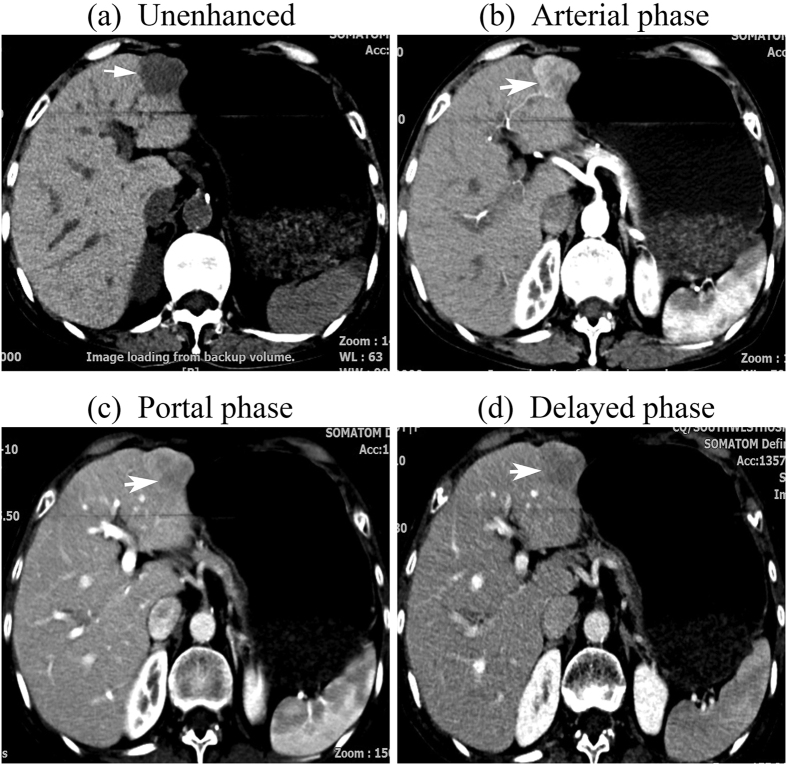
tif CT images of a 69-year-old woman with a 2.7-cm –diameter ICC displaying wash-in and wash-out enhancement pattern. The nodule was hypodense at unenhanced CT (**a**). After intravenous contrast administration, the nodule shows marked hyper-enhancement during the arterial phase (**b**) followed by wash-out during the portal phase (**c**) and the late phase (**d**) (arrow).

The analysis of the vascular dynamic enhancement pattern throughout the different phases of the 98 ICC nodules is shown in Table 3. Twenty eight nodules (28.6%) showed a stable contrast enhancement pattern during the dynamic study including 20 peripherally hyperdens (Fig. 1),6 hypodense and 2 hyperdense during all the three vascular phases. Thirty five nodules (35.7%) demonstrated a progressive enhancement pattern: peripherally hyperdense in the arterial phase followed by centripetal progressive enhancement in the portal and the late phase in 27 nodules (Fig. 2), inhomogeneous hyperdense in the arterial phase followed by progressive enhancement in the portal and the late phase and reached maximal intensity in the delayed phase in 4 nodules, hypodense during the arterial and the portal phase followed by inhomogeneous hyperdense during the late phase in 2 nodules, globally hypodense during the arterial phase followed by peripherally hyperdense during the portal phase and the late phase in 2 nodules. Fifteen nodules (15.3%) displayed a “wash-in and washout” enhancement pattern: intense hyperdense during the arterial phase followed by hypodense during the portal and the late phase in 13 nodules (Fig. 3), intense hyperdense during the arterial phase and the portal phase followed by hypodense during the late phase in 2 nodules. Twenty nodules (20.4%) showed other enhancement patterns: peripherally hyperdense during the arterial phase and the portal phase followed by hypodense during the late phase in 5 nodules, peripherally hyperdense during the arterial phase followed by hypodense during the portal phase and the late phase in 3 nodules, inhomogeneous hyperdense during the arterial phase followed by peripherally hyperdense during the portal phase and inhomogeneous hyperdense during the late phase in 2 nodules, slightly inhomogeneous hyperdense during the arterial phase followed by hypodense during the portal phase and the late phase in 6 nodules, hyperdense during the arterial phase and the portal phase followed by isodense during the late phase in 1nodule, isodense during the arterial phase followed by peripherally hyperdense during portal phase and hypodense during the late phase in 1 nodule, slightly inhomogeneous hyperdense during the arterial phase followed by peripherally hyperdense during the portal phase and the late phase in 1 nodule, peripherally hyperdense during the arterial phase followed by isodense during the portal phase and hypodense during the late phase in 1 nodule.

Enhancement patterns of ICC nodules during dynamic contrast CT scan according to nodule size. There were no significant differences in the dynamic vascular patterns of ICC previously defined according to nodule size (≤3 cm, 3.1–5.0 cm, >5 cm), including stable enhancement (*p* = 0.174, *p* = 0.429, *p* = 0.414, respectively), progressive enhancement (*p* = 0.724, *p* = 0.362 *p* = 0.565, respectively), wash-out enhancement (*p* = 0.718, *p* = 1.000, *p* = 0.807, respectively) and other enhancement patterns (*p* = 0.240, *p* = 0.189, *p* = 1.000, respectively).

### Characterization of nodules according to enhancement pattern at CT

Thirty five nodules demonstrating a progressive enhancement pattern and 28 nodules showing a stable contrast enhancement pattern were characterized as ICC (64.3%). Fifteen nodules displaying a “wash-in and washout” enhancement pattern were characterized as HCC ((15.3%)). Twenty nodules showing other enhancement patterns were indeterminate (20.4%).

## Discussion

Ninety eight ICC consecutively identified in cirrhotic patients between January 2005 and July 2015 in our hospital exhibited varied enhancement patterns at contrast-enhanced multiphase multidetector CT. Over half of the ICC nodules (54.1%) had a peripheral rim-like enhancement during the arterial phase, whereas during the portal and delayed phase, 26 nodules (49.1%) showed a centripetal progressive enhancement,20 nodules (37.7%) a stable enhancement and 7 nodules (13.2%) other enhancement pattern. The proportion of ICC nodules showing peripheral rim-like enhancement during the arterial phase in our patients was similar to previous studies by Kim *et al*.^11^ and Lavarone *et al*. (42.8%, 50% respectively). We consider that the presence of a central necrosis and fibrosis may account for the rim-like arterial contrast uptake at the periphery of the nodules^13^. The rate of ICC nodules showing a progressive enhancement pattern in our study (35.7%) is similar to that reported by Kim *et al*. (32.1%)^11^ but slightly lower than that by Lavarone *et al*. (42%)^14^. We favor to interpret these slight variances in the light of the difference of sample size.

Twenty two ICC nodules (15.3%) displayed a “wash-in and wash-out” enhancement pattern in our study, resembling the radiological hallmarks of HCC, namely, arterial hypervascularity followed by venous or delayed phase washout^10^, and in fact, these nodules were independently characterized as HCC by radiologists with over 20 year experience of liver radiology in our hospital (PC and SYD). The rate of ICC nodules showing “wash-in and wash-out” enhancement pattern in our study is lower than that reported by Kim *et al*. (21.4%)^11^ but higher than that by Galassi *et al*. (4.2%)^12^ and Lavarone *et al*. (0%)^14^. We favor to interpret the difference from that of Kim *et al*. in the light of two reasons: (1) the difference of sample size (28 nodules in the study of Kim *et al*., 98 nodules in the present study); (2) the difference of scanning protocol. All the 26 patients in the study of Kim *et al*. underwent dual-phase helical CT (arterial phase and portal venous phase imaging), without providing any account on their contrast appearance during the delayed phase, reflecting the lack of an internationally accepted, standardized protocol for contrast CT scan in the diagnosis of ICC. An explanation of the difference from that of Galassi *et al*. may be the sample size and etiology of cirrhosis (25 ICC nodules ≤5 cm enrolled and 62.5% of cirrhosis related to hepatitis C virus infection in the study of Galassi *et al*., 48 ICC nodules ≤5 cm enrolled and 85.7% of cirrhosis related to hepatitis B virus infection in our study). The difference from that of Lavarone *et al*. may be attributed to the following three factors: (1) different definition of “wash-in and wash-out” enhancement pattern; (2) the difference in tumor size; (3) the difference of sample size and etiology of cirrhosis. The definition of “wash-in and wash-out” by lavarone *et al*. was global intense contrast enhancement during the arterial phase followed by contrast wash-out in the portal and/or delayed phase^14^. However, this definition may not be suitable for characterizing nodules that show predominantly hyperdense (i.e., involving 60∼90% of the nodule cross section area) during the arterial phase followed by hypodense in the portal and/or delayed phase. The majority of HCC in cirrhosis displayed inhomogeneous enhancement during the arterial phase^11^,^15^,^16^, therefore, it is not reasonable to correspond arterial hypervascularity to only global intense contrast enhancement during the arterial phase. In our opinion, to define predominantly hyperdense of a nodule during arterial phase as arterial hypervascularity, regardless of global or predominantly partial^13^,^15^ is more reasonable. The median tumor size was 3.0 cm in the study of lavarone *et al*.^14^, while 5.3 cm in our study. However, after stratification, the ICC nodules ≤3 cm enrolled in our study (21 nodules) is comparable to that of lavarone *et al*. (23 nodules) because much more ICC nodules were included in our series. More importantly, 4 ICC nodules ≤3 cm (19.0%) showed “wash-in and wash-out” enhancement pattern and there were no significant differences in the dynamic vascular patterns of ICC according to nodule size (≤3 cm, 3.1–5.0 cm, >5 cm) in our study. Patients with cirrhosis who undergo surveillance may have an earlier stage of HCC at diagnosis^17^. Unfortunately, surveillance for HCC in patients with cirrhosis is recommended but may not be successfully performed. Less than 20% of patients with cirrhosis who developed HCC received regular surveillance in the United States^18^. HCCs were detected during surveillance in the minority of patients even at major referral center^19^. Surveillance for HCC is still not a consolidated practice as it should be^20^. Therefore, differentiation of ICC from HCC in large nodules is still a common clinical situation worldwide for most liver tumors in cirrhosis are found not at very early stage. (3) Forty ICC nodules were enrolled and 18.8% of cirrhosis related to hepatitis B virus infection in the study of lavarone *et al*., while 98 ICC nodules enrolled and 85.7% of cirrhosis related to hepatitis B virus infection in our study. Additionally, in the paper of lavarone *et al*., one ICC nodule showed hyperdense during both the arterial and portal phases, followed by hypodense in the delayed venous phase (patient No. 33, Table 3), should be judged as “wash-in and wash-out” pattern according to the definition, though wash-out occurred in the delayed phase and be subsequently characterized as HCC. Because radiological diagnosis of HCC should be based on imaging techniques (4-phase MDCT/dynamic contrast enhanced MRI) showing arterial hypervascularity and venous or delayed phase washout according to the practice guidelines of AASLD and EASL^9^,^10^. This discrepancy reflects the lack of internationally agreed standard for the review of radiological films aimed at optimizing the diagnosis of ICC in cirrhosis. Our results indicate if dynamic contrast CT scan was used as the sole modality for the non-invasive diagnosis of HCC in cirrhosis, about 15% of ICC would be misdiagnosed for HCC, however, this estimate needs to be clarified prospectively in the future.

There were some limitations to our study. First, it was limited by its retrospective nature and thus by our limited control regarding patient selection. Therefore, we are unable to calculate the sensitivity and specificity of dynamic contrast CT in differentiation between ICC and HCC in cirrhosis. Second, though we provided a largest series of pathologically proven ICC in cirrhosis seen on contrast-enhanced multiphasic multidetector CT up to now, the sample size of ICC nodules ≤3 cm is still limited and just comparable to that of the previous study^14^. This reflects the low incidence of ICC in patients with cirrhosis, accounting for about 1–2% of all new nodules in cirrhosis^21^,^22^ and surveillance for hepatic tumor in patients with cirrhosis is not widely performed in our country. Prospective multiple center study including more small ICCs in cirrhosis is expected Third, it is impossible for us to compare the enhancement patterns of CT and MRI because only 11 out of 84 patients of ICC underwent both MRI and CT scan in our series, though the enhancement appearance of ICC at MRI was reported much different from that of HCC^23^ and this reflects the fact that in many countries worldwide, with a high incidence of HCC, MRI is still not as readily available as CT. The usefulness of our present results is restricted to centers which use CT for characterization of hepatic nodules in cirrhosis.

In conclusion, ICC in cirrhosis has varied enhancement patterns at contrast-enhanced multiphase multidetector CT. Though the majority of ICC did not display typical radiological hallmarks of HCC, if dynamic CT scan was used as the sole diagnostic modality for the non-invasive diagnosis of nodules in cirrhosis, the risk of misdiagnosis of ICC for HCC is not negligible.

## Additional Information

**How to cite this article**: Li, R. *et al*. Dynamic enhancement patterns of intrahepatic cholangiocarcinoma in cirrhosis on contrast-enhanced computed tomography: risk of misdiagnosis as hepatocellular carcinoma. *Sci. Rep*. **6**, 26772; doi: 10.1038/srep26772 (2016).

## Figures and Tables

**Table 1:** Demography of 84 patients with ICC in cirrhosis.

Age median[range] (years)	50 [24 ～ 75]
Gender, male/female, No (%)	66 (78.6)/18 (21.4)
Etiology of cirrhosis, No (%)	
HBV	52 (61.9)
HBV + alcohol	20 (23.8
HCV	0
Alcohol	3 (3.6)
Biliary	1 (1.1)
unknown	8 (9.5)
Diabetes, No (%)	7 (8.3)
Cigarette smokers, No (%)	39 (46.4)
Child-Pugh class A, No (%)	75 (89.3)
AFP (ng/ml) median[range] (ng/ml)	6.3[0.2 ～ 5940.0]
≤20 ng/ml, No (%)	65 (77.4%)
>20 ng/ml, No (%)	19 (22.6)
CA19-9 (U/ml) median[range] (U/ml)	33.5[2.0 ～ 745.7]
≤37 U/ml, No (%)	41 (48.8%)
>37 U/ml, No (%)	43 (51.2%)
Number of nodules	
Single nodule, No (%)	75 (89.3)
Multiple nodule, No (%)	9 (10.7)
Nodule size median[range] (cm)	5.3[1.0 ～ 13.6]
≤3 cm, No (%)	21 (20.4)
3.1–5.0 cm, No (%)	27 (27.6)
>5 cm, No (%)	50 (51.0)

No: number; HBV: hepatitis B virus; HCV: hepatitis C virus; AFP: alpha-fetoprotein.

CA 19-9: carbohydrate antigen 19-9.

**Table 2:** Contrast-enhanced CT findings of 98 ICC nodules according to the different vascular enhancement phases.

	Arterial phase	Portal phase	Delayed phase
Hyperdense No. (%)	35 (35.7)	13 (13.3)	15 (15.3)
Peripherally hyperdense No. (%)	53 (54.1)	53 (54.1)	51 (52.0)
Isodense No. (%)	1 (1.0)	1 (1.0)	1 (1.0)
Hypodense No. (%)	9 (9.2)	31 (31.6)	31 (31.6)

**Table 3:** Dynamic enhancement patterns of 98 ICC nodules at CT according to tumor size.

Enhancement pattern	≤3c (n = 21)	3.1–5.0 (n = 27)	>5 cm (n = 50)
Stable enhancement	4	10	14
Progressive enhancement	6	9	20
Wash-in and wash-out	4	3	8
Other patterns	7	5	8
